# The effects of coenzyme Q_10_ supplement on blood lipid indices and hepatic antioxidant defense system in SD rats fed a high cholesterol diet

**DOI:** 10.1186/s42826-019-0013-1

**Published:** 2019-08-08

**Authors:** Ha-Na Kim, Dong-Gyung Jeon, Yong Lim, In-Surk Jang

**Affiliations:** 1 Department of Animal Science and Biotechnology, and the Regional Animal Research Center, Gyeongnam National University of Science and Technology, Chilam-Dong 150, Jinju, Gyeongnam 52725 Korea; 2 Department of Clinical Laboratory Science, Dong-Eui Univerisity, Busan, 47340 Korea

**Keywords:** Coenzyme Q_10_, Cholesterol, Antioxidant enzymes, Lipid indices

## Abstract

A total of 24 SD rats were allotted to four treatment groups such as the control (CON), 1% of cholesterol diet (CHO), 0.5% of coenzyme Q_10_ (COQ) and 1% of cholesterol plus 0.5% of coenzyme Q_10_ (CHCQ) groups to determine the effects of coenzyme Q_10_ (CoQ_10_) on the antioxidant defense system in rats. The body weight, weight gain, liver weight and abdominal fat pads were unaffected by 0.5% of CoQ_10_ supplement in the rats. The level of triglyceride and HDL-cholesterol levels in the blood was significantly increased (*p* < 0.05) by the 1% of cholesterol supplement (CHO), whereas 0.5% of CoQ_10_ supplement (COQ) did not alter these blood lipid indices. In the mRNA expression, there was a significant effect (*P* < 0.05) of the CoQ_10_ supplement on the mRNA expression of superoxide dismutase (SOD), although the mRNA expression of glutathione peroxidase (GPX) and glutathione S-transferase (GST) was unaffected by cholesterol or the CoQ_10_ supplement. Similar to mRNA expression of SOD, its activity was also significantly increased (*P* < 0.05) by CoQ_10_, but not by the cholesterol supplement effect. The activities hepatic GPX and GST were unaffected by CoQ_10_ and cholesterol supplements in rats. Lipid peroxidation in the CHO group resulted in a significant (*p* < 0.05) increase compared with that in the other groups, indicating that the CoQ_10_ supplement to 1% of cholesterol-fed rats alleviated the production of lipid peroxidation in the liver. In conclusion, 0.5% of the CoQ_10_ supplement resulted in positive effects on the hepatic antioxidant defense system without affecting blood lipid indices in 1% of cholesterol fed rats.

## Introduction

Coenzyme Q_10_ (CoQ_10_) is a fat soluble component primarily synthesized by the mitochondria of the heart, liver, kidneys, pancreas and muscles, where it is consumed for a large quantity of ATP production [1, 2]. It has been also reported that CoQ_10_ as an antioxidant in the phospholipid bilayer of cell membranes ameliorate metabolic disease such as cardiovascular disease, neurodegenerative disease, mitochondrial disorder and diabetes in humans [3–5]. In human studies, insufficient CoQ_10_ synthesis is a common disorder in ageing conditions due to the process of cellular aging, especially in elderly people [5]. Numerous studies have demonstrated that dietary CoQ_10_ supplement significantly alleviates various degenerative diseases and toxicity via activating the antioxidant defense system in human and rodents [1, 6, 7]. In particular, dietary supplementation with CoQ_10_ has been recommended to improve clinical and metabolic disorder for cardiovascular disease, especially in abnormal conditions such as ageing and oxidative stress [3, 8]. Especially because of the vital role of CoQ_10_ in antioxidant defense system, it has been suggested that dietary CoQ_10_ may protect against oxidative damage induced by reactive oxygen species (ROS), which is produced under certain physiological conditions [6, 9]. In animal studies, CoQ_10_ intake appeared to increase in antioxidative potential of tissues [10]. However, several studies insisted that the endogenous production level was enough to provide sufficient CoQ_10_ to prevent deficiency in young healthy animals [11, 12].

At present, the effectiveness of oral supplementation of CoQ_10_ varies greatly depending upon the physiological and metabolic status of animals, health of individuals, level and period of CoQ_10_ supplement, etc. [1, 11, 13]. Recently, it has been reported that antioxidant therapy with CoQ_10_ may have beneficial effects associated with atorvastatin-induced myopathy in hyperlipidemic rats [14]. Atorvastatin, a member of the drug class known as statins used for lowering cholesterol, also inhibit the synthesis of CoQ_10_ in the body, because CoQ_10_ and cholesterol are both synthesized from the same precursor known as mevalonate. This harmful effect might be overcome by CoQ_10_ supplement to protect cell integrity against reactive oxygen species (ROS) and lipid peroxidation induced by statin agents [14]. Despite rather well-recognized antioxidant effects of CoQ_10_ in vitro*,* the effectiveness of CoQ_10_ as an antioxidant against oxidative stress varies greatly depending on the study. Thus, it is still controversial whether the supplementation of CoQ_10_ can affect the antioxidant system in vivo, although numerous research studies have been conducted.

To explore the effects of CoQ_10_ on the antioxidant defense mechanism under the circumstance of oxidative stress in our study, therefore, we applied a CoQ_10_ supplement to the rats fed a high cholesterol diet. Since involvement of oxidative stress induced by high cholesterol in the pathological damage of the heart, liver and arteries has been well studied, it can be hypothesized that feeding 1–2% cholesterol to rats might lead to an increase in cholesterol-mediated tissue damage occurred by lipid peroxidation [15–17].

In the context of the described reasons, the objective of the study was to determine whether a dietary supplement of CoQ_10_ could affect the blood lipid indices, expression of antioxidant enzymes and antioxidant status in SD rats fed a 1% of cholesterol diet.

## Materials and methods

### Experimental animals and design

The animal experiment was approved by the Institutional Animal Care and Use Committee (IACUC, No 201509) at the Gyeongnam National University of Science and Technology in Korea. Male Sprague Dawley (SD) rats aged 5-wks were obtained from Samtako (Osan, Korea). After an adaption of 1-wk period, a total of 24 rats having a similar body weight were allocated to four treatments with free access to lab chow and sterilized water ad libitum in an environmentally controlled room (22 ± 2 °C) with a 12 h light/dark cycle. Rats in group 1 (CON) were fed the control diet (powder type of purified diet mixed with AIN-93G formulation), rats in group 2 (CHO) were fed the control diet supplemented with 1% cholesterol; rats in group 3 (COQ) were given the control diet supplemented with 0.5% CoQ_10_; and rats in group 4 (CHCQ) were given the control diet supplemented with 1.0% cholesterol plus 0.5% CoQ_10_, respectively. Weekly body weight and daily feed intake were recorded throughout a 4-week experimental period. A commercial CoQ_10_ was purchased from Inter Monglia Kingdomway Pharmaceutical Limited (CoQ_10_ 99.3%, Xiamen, China). The CoQ_10_ supplement was dissolved in soybean oil carrier.

### Experimental procedures

#### Tissue harvesting

After 5-week feeding trial, 24 rats were deprived of diet for 12 h and then the rats (*n* = 6) were sacrificed with CO_2_ gas. Immediately after anaesthetizing blood was collected in tubes coated with sodium heparin by heart puncture and plasma was harvested. The liver tissues and abdominal fat pads were collected, weighed and then rapidly frozen in liquid N_2_. All tissues were stored at − 70 °C until further assay.

### Plasma lipid composition analyses

Plasma triglyceride (TG), total cholesterol (TC), HDL-cholesterol (HDL-C) and LDL-cholesterol (LDL-C) were assayed using a clinical biochemical analyzer (Mindray, BS-120, Mindry Bio Medical Electronics co., Shnzhen, China). All analyses were conducted in duplicate.

### mRNA expression of antioxidant genes by RT-PCR

The mRNA expression of antioxidant genes including superoxide dismutase (SOD), glutathione peroxidase (GPX) and glutathione S-transferase (GST) was quantified by real-time PCR (Bio-Rad, CA, USA). Total RNA of liver tissues was extracted using RNeasy plus mini kit (Qiagen, Nordrhein-Westfalen, Germany). Briefly, 650 μL of RPE buffer solution was added to 30 mg of liver tissues homogenized using liquid nitrogen. After spinning down, 600 μL of supernatant was transferred a micro-tube and centrifuged for 30 s. Next, the extracted aqueous solution was mixed with 650 μL of 50% ethanol, after which the mixed solution was centrifuged (15 s at 10,000×*g*). Seven hundred microliter of RW1 buffer was added and then centrifuged (15 s at 11,000×*g*). The precipitated pellet was then washed with RPE buffer and diluted with 40 μL of RNeasy-free water, after which the optical density at 260/230 nm was applied to quantify the concentration of RNA (Gene Quant *pro* spectrophotometer, Amersham, Piscataway, NJ. USA).

The cDNA primers used to amplify antioxidant genes and β-actin are shown in Table 1. For reverse transcription, 1 μg of total RNA were incubated with 1.0 μg of oligo dT (Promega Co., Wisconsin, USA) at 70 °C for 5 min and 4 °C for 5 min to produce the first strand cDNA. The reaction mixture was incubated at 42 °C for 50 min, 90 °C for 10 min and 42 °C for 50 min in a reaction cocktail containing 5× first strand buffer, 2.5 mM dNTP, 0.1 M DTT, superscript III and RT-mixture. The mRNA quantification of antioxidant genes was analyzed by real-time quantitative PCR with SYBR green supermix (Bio-Rad, CA, USA) under the following conditions: 5 min at 95 °C, followed by 40 cycles of denaturation at 95 °C for 15 s and annealing at 60 °C for 30 s and then extension at 72 °C for 30 s. The PCR amplification cycle at which dye fluorescence passed the selected baseline (Ct) was determined by real-time monitoring. The expression of all mRNAs was calculated by the 2^[ΔΔ]^ method [18] to see relative changes in gene expression using β-actin as an internal control.

**Table 1:** Primers used for the quantification of mRNA using real time-PCR

Genes	Primer sequences	Product size (bp)	Gene bank Accession No.
SOD	5′-ACT TCG AGC AGA AGG CAA GC- 3′ 5′-GTC TCC AAC ATG CCT CTC TTC AT- 3′	194	NM017050.1
GPX	5′- CAG TTC GGA CAT CAG GAG AAT −3′ 5′- AGA GCG GGT GAG CCT TCT − 3′	139	NM030826.3
GST	5′-CAAGTCCACTTGTGTGAGTG − 3′ 5′-CAGCTGGACTACCTGAGTTC- 3′	230	L294427.1
β-Actin	5′-GGC ACCACACTTTCTACAAT- 3′ 5′-AGGTCTCAAACATGATCTGG- 3’	123	NM_031144

### Antioxidant enzyme activity, lipid peroxidation and total antioxidant power

The method of Kupfer and Levin [19] was applied to harvest cytosol and microsome fractions of liver tissues. In brief, liver tissues (1 g) were homogenized with a solution containing 0.25 M sucrose, 0.05 M Tris-HCl (pH 7.4), 0.005 M MgCl_2_, 0.025 M KCl and 0.008 M CaCl_2_ using a tissue grinder (Omni TH tissue homogenizer, Omni Int. NW Kennesaw, GA, USA). After then, the homogenate was centrifuged at 10,000×*g* for 15 min, after which time the resulting supernatant was diluted 1: 6 volume with a solution composed of 0.0125 M sucrose, 0.005 M MgCl_2_, 0.025 M KCl and 0.008 M CaCl_2_. The diluted supernatant was centrifuged at 1500×*g* for 10 min, after which time the resulting supernatant was harvested as cytosol fraction. The pellet was dispersed in 0.25 M sucrose was centrifuged at 1500×*g* for 10 min. After that the remnant pellet was suspended in a cold 1.15% KCl solution to harvest a microsomal fraction. The harvested supernatant (cytosol) and a suspended pellet (microsomes) were frozen in liquid nitrogen and stored at − 70 °C until further assay. In brief, SOD activity in the cytosol fraction was measured using a commercial SOD assay kit (Sigma-Aldrich, St. Louis, MO, USA) based on an indirect assay method of xanthine oxidase as described in the manufacture’s protocol. The activity of SOD is presented as units/mg of proteins, where 1 unit of activity was the amount of enzyme required to inhibit 50% of the SOD or SOD like substances. GPX was measured at 37 °C in the cytosol with cumene hydroperoxide as a substrate [20]. The GPX coupled the reduction of cumene hydroperoxide to the oxidation of NADPH by glutathione reductase, and concomitant oxidation was monitored in a spectrophotometer with the decrease in absorbance at 340 nm. One unit of GPX was expressed as the amount of GPX needed to oxidize 1 μM of NADPH per min. Cytosolic GST was determined with 1-chloro-2, 4-dinitrobenzene (CDNB) as a substrate by monitoring the increase in absorbance at 340 nm [21]. One unit of activity was expressed as the amount of enzyme catalyzing the conjugated CDNB per min. The level of lipid peroxidation in the microsome was assayed by measuring amount of 2-thiobarbituric acid (TBA) reactive substances with a spectrophotometer at 532 nm [22]. TBA material is expressed as nanomoles of malondialdehyde (MDA) per milligram of protein. Protein was assayed by the BCA method (Pierce Assay) using an ELISA (V_Max_, Molecular Devices, CA, USA). The total antioxidant power (TAP) in the plasma was measured using a commercially available assay kit with an ELISA reader (Oxford Biomedical Research, Inc. MI, USA). Assay procedures were carried out according to the manufacturer’s protocols. Trolox was used to generate a standard curve, and data were presented as mM Trolox equivalents or in μM copper reducing equivalents.

### Statistical analysis

All values are expressed as means±standard deviation (SD). Statistical analyses were performed using the Proc GLM procedure to analyze two-way analysis of variance models (SAS Institute Inc.). When the treatment effect was significant at *p* < 0.05, Tukey’s multiple comparison test was used to assess significant differences among groups. A *p* value of < 0.05 was considered statistically significant.

## Results

### Growth performance and organ weights

The effects of dietary CoQ_10_ and cholesterol on growth performance and the relative organ weights of SD rats are presented in Tables 2 and 3, respectively. The body weight, gain and feed conversion ratio of rats were unaffected by dietary supplementation with 1.0% of cholesterol or 0.5% of CoQ_10_, although the CHCQ group had a tendency for increased body weight and gain after 4-wks of the experimental period (Table 2). The relative weights of the liver and abdominal fat were significantly increased (*p* < 0.05) only by the cholesterol supplement, resulting that the CHCQ group showed higher (*p* < 0.05) liver and abdominal fat pad weights compared with the CON rats (Table 3).

**Table 2:** Effect of coenzyme Q_10_ supplement on body weight, gain, feed intake and feed conversion ratio (FCR) in SD rats fed 1% of cholesterol diet

Items	Treatment						
	No cholesterol		Cholesterol (1%)		Significance		
	No CoQ_10_	CoQ_10_ (0.5%)	No CoQ_10_	CoQ_10_ (0.5%)			
	CON	COQ	CHO	CHCQ	Coq	Cho	C*C
Initial BW (g), 7 wks	183.96 ± 6.84	184.93 ± 7.25	184.42 ± 6.78	186.20 ± 7.24	NS	NS	NS
Final BW (g), 11 wks	389.55 ± 11.90	380.88 ± 28.25	379.83 ± 10.75	402.35 ± 20.49	NS	NS	NS
Weight gain (g)	205.59 ± 14.4	195.95 ± 23.31	195.41 ± 14.96	216.15 ± 16.55	NS	NS	^*^
Feed intake (g/day)	20.37 ± 1.38	22.16 ± 0.82	21.17 ± 2.79	23.49 ± 1.68	NS	NS	NS
FCR	3.48 ± 0.30	4.00 ± 0.37	3.79 ± 0.40	3.82 ± 0.38	NS	NS	NS

Means±SD (*n* = 6)

*NS* indicates non-significant, *Coq* effect of coenzyme Q_10,_ *Cho* effect of cholesterol, C*C interaction effect between coenzyme Q_10_ and cholesterol

^*^Indicates significant difference at *p* < 0.05

**Table 3:** Effect of coenzyme Q_10_ supplement on the weights of the liver and abdominal fat in SD rats fed 1% of cholesterol diet

Items	Treatment						
	No cholesterol		Cholesterol (1%)		Significance		
	No CoQ_10_	CoQ_10_ (0.5%)	No CoQ_10_	CoQ_10_ (0.5%)			
	CON	COQ	CHO	CHCQ	Coq	Cho	C*C
Liver weight, g	11.17 ± 0.94^bc^	10.41 ± 0.84^c^	12.27 ± 0.56^ab^	13.57 ± 0.98^a^	NS	^*^	^*^
Abdominal fat pad, g/100 g BW	1.35 ± 0.49^b^	1.81 ± 0.14^ab^	2.00 ± 0.41^a^	1.96 ± 0.35^a^	NS	^*^	NS

Means±SD (*n* = 6) with different superscript differ among groups (*p* < 0.05)

*NS* indicates non-significant, *Coq* effect of coenzyme Q_10,_ *Cho* effect of cholesterol, C*C interaction effect between coenzyme Q_10_ and cholesterol

^*^Indicates significant difference at *p* < 0.05

### Blood lipid indices and total antioxidant power

The plasma lipid components including TG and HDL-cholesterol were significantly increased (*p* < 0.05) by 1% of the cholesterol supplement, whereas 0.5% of the CoQ_10_ supplement did not affect these lipid indices (Table 4). The CHO rats fed 1% of the cholesterol diet exhibited a significant increase (*P* < 0.05) in triglyceride level compared with the other groups. The CON and COQ groups fed no cholesterol diet showed a significantly higher (*p* < 0.05) HDL-C compared with the CHO and CHCQ groups fed 1% of cholesterol. The CHO group also showed much higher (*P* < 0.05) ratios of TC/HDL-C and LDL-C/HDL-C than the CON group, indicating that 1% of cholesterol supplement to diet resulted in a greater the atherogenic risks in rats. However, 0.5% of CoQ_10_ supplement did not improve the ratio of these indicators (Table 4).

**Table 4:** Effect of coenzyme Q_10_ supplement on blood lipid profiles in SD rats fed 1% of cholesterol diet

Items	Treatment						
	No Cholesterol		Cholesterol (1%)		Significance		
	No CoQ_10_	CoQ_10_ (0.5%)	No CoQ_10_	CoQ_10_ (0.5%)			
	CON	COQ	CHO	CHCQ	Coq	Cho	C*C
Triglyceride (mg/dl)	41.00 ± 15.02^b^	39.40 ± 17.33^b^	72.00 ± 22.95^a^	43.60 ± 8.26^b^	NS	^*^	NS
Total cholesterol (mg/dl)	140.04 ± 19.79	144.10 ± 9.36	123.86 ± 17.23	131.56 ± 11.94	NS	NS	NS
HDL-cholesterol (mg/dl)	94.72 ± 15.24^a^	100.62 ± 6.01^a^	67.80 ± 15.28^b^	77.68 ± 6.11^b^	NS	^*^	NS
LDL-cholesterol (mg/dl)	37.12 ± 9.97	35.60 ± 7.02	41.66 ± 8.16	45.16 ± 7.27	NS	NS	NS
TC/HDL ratio	1.48 ± 0.10^b^	1.44 ± 0.10^b^	1.86 ± 0.26^a^	1.69 ± 0.08^a^	NS	^*^	NS
LDL/HDL ratio	0.40 ± 0.08 ^b^	0.36 ± 0.07^b^	0.64 ± 0.21^a^	0.58 ± 0.09^a^	NS	^*^	NS

Means±SD (*n* = 6) with different superscript differ among groups (*p* < 0.05).

*NS* indicates non-significant, *TC/HDL* ratio indicates ratio of total cholesterol to HDL-cholesterol, *LDL/HDL* ratio indicates ratio of LDL-cholesterol to HDL-cholesterol, *Coq* effect of coenzyme Q_10,_ *Cho* effect of cholesterol, C*C interaction effect between coenzyme Q_10_ and cholesterol

^*^Indicates significant difference at *p* < 0.05

The plasma TAP level was not altered by the cholesterol or CoQ_10_ supplement, although there was a numerically increased TAS in the COQ group (Table 5).

**Table 5:** Effect of coenzyme Q_10_ supplement on the total antioxidant power (TAP) in the plasma of SD rats fed 1% of cholesterol diet

Items	Treatment						
	No cholesterol		Cholesterol (1%)		Significance		
	No CoQ_10_	CoQ_10_ (0.5%)	No CoQ_10_	CoQ_10_ (0.5%)			
	CON	COQ	CHO	CHCQ	Coq	Cho	C*C
	Umoles Trolox equivalents						
TAP	127.96 ± 18.17	135.93 ± 18.16	127.96 ± 28.43	141.72 ± 10.24	NS	NS	NS

Means±SD (*n* = 6)

*NS* indicates non-significant, *Coq* effect of coenzyme Q10, *Cho* effect of cholesterol, C*C interaction effect between coenzyme Q10 and cholesterol

### mRNA expression and activity of hepatic antioxidant genes and lipid peroxidation

**Fig. 1:**
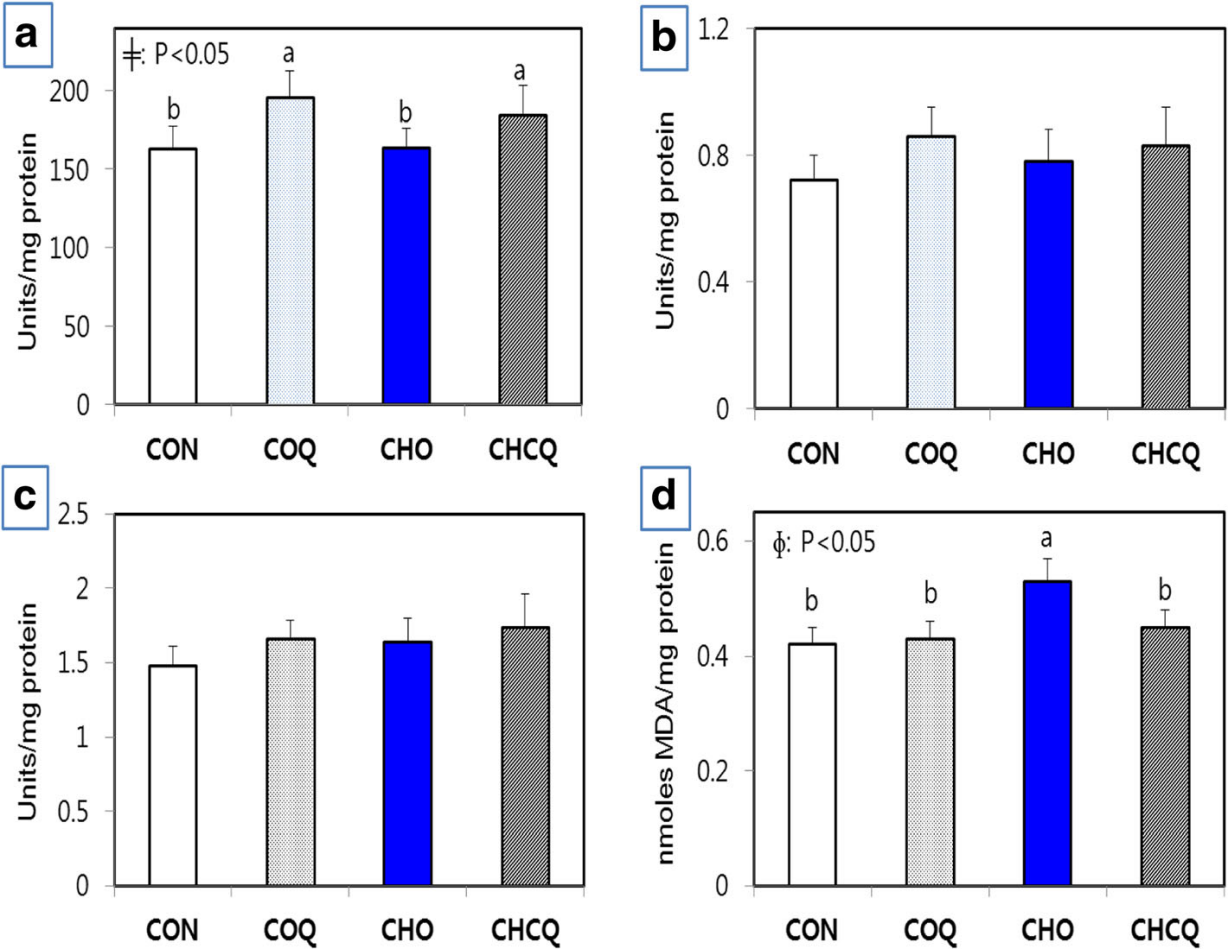
Specific activity of antioxidant enzymes (**a**: SOD, **b**: GPX and **c**: GST) and lipid peroxidation (**d**) in the liver of SD rats designated as the control (CON), 1% of cholesterol diet (CHO), 0.5% of CoQ_10_ (COQ) and 1% of cholesterol plus 0.5% of CoQ_10_ (CHCQ). Means (Mean ± SD, *n* = 6) with different superscript differ among groups (*p* < 0.05). ɸ indicates the significance effect (*P* < 0.05) of coenzyme Q_10_ supplement. ╪ indicates the significance effect (*P* < 0.05) of cholesterol supplement

The effects of the cholesterol and CoQ_10_ supplement on the mRNA expression and activity of SOD, GPX and GST are shown in Table 6 and Fig. 1, respectively. In the mRNA expression, there was a significant increase (*P* < 0.05) in SOD in response to CoQ_10_ supplement, although the mRNA expression of GPX and GST was not affected by the supplement with cholesterol or CoQ_10_ (Table 6). In the specific activity of antioxidant enzymes, SOD activity was also significantly up-regulated (*P* < 0.05) by the CoQ_10_ supplement, but not by the cholesterol supplement (Fig. 1). The CoQ_10_ supplemented groups showed a significantly (*p* < 0.05) higher activity of SOD than the CON and CHO groups. In particular, the supplementation with CoQ_10_ to rats fed 1% of the cholesterol diet showed a significant increase in SOD activity, which was comparable to the COQ rats (Fig. 1). Dietary supplement with CoQ_10_ resulted in a significant increase in the mRNA expression and activity of SOD. The activities of GPX and GST were not altered by the administration of CoQ_10_ or cholesterol in the liver of SD rats (Fig. 1), which was similar to the mRNA expression pattern of these genes.

**Table 6:** Effect of coenzyme Q_10_ supplement on the mRNA expression of antioxidant enzymes (SOD, GPX and GST) in the liver of SD rats fed 1% of cholesterol diet

Items	Treatment										
	No Cholesterol				Cholesterol (1%)				Significance		
	No CoQ_10_		CoQ_10_ (0.5%)		No CoQ_10_		CoQ_10_ (0.5%)				
	CON		COQ		CHO		CHCQ		Coq	Cho	C*C
	Δ Ct	2^-ΔΔCt^	Δ Ct	2^-ΔΔCt^	Δ Ct	2^-ΔΔCt^	Δ Ct	2^-ΔΔCt^			
SOD	7.62 ± 1.69	1	6.02 ± 1.56	3.03	7.13 ± 1.54	1.40	6.12 ± 0.54	2.83	^*^	NS	NS
GPX	3.52 ± 2.15	1	2.01 ± 1.58	2.86	3.26 ± 1.05	1.20	3.27 ± 1.08	1.20	NS	NS	NS
GST	14.39 ± 2.08	1	14.64 ± 2.12	0.84	12.50 ± 1.42	3.71	15.43 ± 2.62	0.48	NS	NS	NS

Means±SD (*n* = 6)

The values are ∆Ct, which is represented as the Ct of each target gene corrected by Ct of the control gene (β-actin)

The fold difference in the relative expression of the target gene was calculated as the 2^-∆∆Ct^

*NS* indicates non-significant, *Coq* effect of coenzyme Q_10,_ *Cho* effect of cholesterol, C*C interaction effect between coenzyme Q_10_ and cholesterol

^*^Indicates significant difference at *p* < 0.05

Lipid peroxidation in the CHO group resulted in a significant (*p* < 0.05) increase compared with that in the CON and COQ groups (Fig. 1d). However, CoQ_10_ supplement to 1% of cholesterol-fed rats normalized the level of lipid peroxidation, which was comparable to that of the CON rats. This result indicated that the hepatic lipid peroxidation was ameliorated by 0.5% of the CoQ_10_ supplement in rats fed 1% of the cholesterol diet.

## Discussion

The present study was carried out to investigate whether a dietary supplement of CoQ_10_ could affect performance, organ weight, the blood lipid indices and expression of antioxidant enzymes in cholesterol diet-induced hyperlipidemic rats. The body weight gain and relative weights of the liver and abdominal fat were not influenced by the CoQ_10_ supplement. These results were in agreement with the previous study [8], which reported that the CoQ_10_ did not affect animal performance and organ weights in the SD rats. As expected, it is well known that high-cholesterol diet can increase in the weights of body, liver and abdominal fat pads, as well as blood cholesterol and triglyceride levels [23, 24]. In this study, 1% of the cholesterol diet resulted in a significant increase in the weights of abdominal fat and liver, and blood triglyceride, and a decrease in blood HDL-cholesterol without affecting body weight and blood total cholesterol. We calculated the ratios of TC/HDL-C and LDL-C/HDL-C to see the predictive parameters of lipidemic stress, which are known as the most crucial indices of the atherogenic risks rather than each isolated parameters [25]. It is recognized that the higher ratio of these parameters is, the greater the atherogenic risks [26]. In our study, the rats fed 1% of the cholesterol diet showed much higher ratios of TC/HDL-C and LDL-C/HDL-C than the control rats, indicating that 1% of the cholesterol diet induced a greater lipidemic stress in SD rats.

Our data was in agreement with the results of Ramlho et al. [24], which reported that high fat diet resulted in a changes in the blood level of triglyceride, HDL-C and visceral fat without affecting body weight. In addition, the CoQ_10_ supplement to rats fed 1% of the cholesterol diet significantly alleviated cholesterol-induced increases in blood triglyceride and the relative liver weight of rats. However, we did not see a significant improvement in the ratios of TC/HDL-C and LDL/HDL-C in response to the dietary CoQ_10_ supplement. In partial agreement with our results, several studies with animals have suggested that CoQ_10_ significantly decreased blood triglyceride in a high fat diet fed rats or hepatic lipid concentration with no effect on plasma lipid components in atherogenic rabbits [27, 28]. However, a study with humans [29] demonstrated that a CoQ_10_ supplement to patients with hyperlipidemia improved serum HDL-cholesterol as well as the ratios of TC/HDL-C and LDL/HDL-C, suggesting that dietary CoQ_10_ might be associated with a reduction in the risks of myocardial infarction in patients.

CoQ_10_, a ubiquinone redox-active lipophilic antioxidant which is synthesized in the phospholipid membrane of the tissues, seems to play a crucial role in antioxidant system in various organs of animals. Among various organs, the liver is the major organ of cholesterol metabolism including biosynthesis, storage, excretion and converting cholesterol to bile acids under normal circumstances [30]. Therefore, it is necessary to explore the effects of CoQ_10_ on the expression of antioxidant genes and lipid peroxidation in the liver of high cholesterol diet fed rats. In the present study, there was a significant effect of CoQ_10_ supplement on the induction of mRNA expression and activity of SOD in rats. It was also observed that CoQ_10_ significantly reduced the lipid peroxidation level in rats fed a CoQ_10_ supplemented diet. Thus, the alleviative effects of CoQ_10_ against hepatic oxidative stress in the liver of rats fed 1% of a cholesterol diet may be associated with an antioxidant defense mechanism via increasing SOD expression. A significant increase in SOD in response to CoQ_10_ supplement converts superoxide anion to hydrogen peroxide in a cellular antioxidant reaction, leading to a decrease in the hepatic lipid peroxidation of rats. Thus, it could be postulated that the supplementation with CoQ_10_ might help in the maintenance of the hepatic cellar integrity of the liver against high cholesterol-induced lipidemic stress in SD rats. In the antioxidant defense mechanism, both the non-enzymatic innate free radical scavengers including glutathione and CoQ_10_ and the endogenous antioxidant enzymes such as SOD, GPX and GST play an important functional role in cells to protect cellular membrane against oxidative stress [31].

Several studies reported that CoQ_10_ protected hepatic cellar membranes against ROS produced by oxidative stress under metabolic processes [4, 6, 32–34]. In high fat and cholesterol induced hyperlipidemia of rabbits, a supplementation with CoQ_10_ decreased in mitochondrial ROS and DNA damage in the liver [27]. This results was supported by the in vitro study that dietary CoQ_10_ supplement significantly reduced ROS production and a subsequent improvement of mitochondrial functions [9].

Rats that received a CoQ_10_ supplemented diet (150 mg CoQ_10_/kg/d) showed an increased mitochondrial CoQ_10_ level and antioxidant potential and decreased protein oxidative damage [10]. This study also demonstrated that 0.5% of a CoQ_10_ supplement (equivalent to 100 mg/day) had a potential antioxidant effect against cholesterol-induced oxidative stress in rats. Some literature also pointed out that a beneficial effect of CoQ_10_ on the antioxidant defense system was observed in animals exposed to toxic substances such as lipopolysaccharide (LPS) [4, 33, 35], although the exact mechanism by which dietary CoQ_10_ provides a beneficial effect still remain. In partial agreement with our study, dietary CoQ_10_ resulted in a significant increase in the activities of SOD and CAT in alloxan-induced diabetic rats [6]. Our previous study also demonstrated that supplementation with CoQ_10_ in rats challenged with LPS maintained the same level of SOD, GPX and lipid peroxidation compared with normal rats.

However, it remains controversial whether supplemented CoQ_10_ can increase in the tissue deposition of CoQ_10_ and antioxidant capacity, since endogenous biosynthesis of CoQ_10_ was enough to maintain metabolic process under normal conditions [36]. Several studies reported that the beneficial effects of dietary CoQ_10_ were only proven under abnormal physiological status including ageing, metabolic disorder, exposure of prooxidant substances [1, 3, 36]. Contradictory studies on the effects of CoQ_10_ on the antioxidant defense system of the mitochondria have been published. A study suggested that CoQ_10_ supplement might directly contribute to the antioxidant defense system via potentiating the electron transport chain in the mitochondria of the liver [36], rather than modulating the expression of antioxidant enzymes in animals. They insisted that dietary CoQ_10_ did not directly affect the expression of antioxidant enzymes in tissues [11, 37].

According to the literatures to date, it seems that the antioxidant effects of CoQ_10_ vary depending on the physiological status of animals, dosage and duration of CoQ_10_ level, environmental conditions, etc. [6, 9, 11, 38], although CoQ_10_ is known to maintain the hepatic antioxidant function via the modulation of antioxidant enzymes or its antioxidant capacity under oxidative stress.

## Conclusions

In the study, a dietary CoQ_10_ supplement appeared to have potentiating effects on the antioxidant defense system by the direct induction of hepatic SOD expression in rats fed a 1% of cholesterol diet. Therefore, it could be concluded that dietary CoQ_10_ had beneficial effects on the hepatic antioxidant defense system under cholesterol-induced hyperlipidemic stress in SD rats.

## Data Availability

The datasets generated during the current study are available from the corresponding author on reasonable request. The datasets are available in the data sheet of In-Surk Jang repository.
